# The role of social capital in shaping livelihood for rural Vietnamese households

**DOI:** 10.1371/journal.pone.0295292

**Published:** 2023-12-14

**Authors:** Huynh Ngoc Chuong

**Affiliations:** University of Economics and Law and Vietnam National University, Ho Chi Minh City, Vietnam; The University of Hong Kong, HONG KONG

## Abstract

This study explores the role of social capital in the livelihoods of rural households in Vietnam, examining both direct and indirect impacts. The author uses a revised sustainable livelihood framework to analyze social capital, focusing on bonding-bridging and linking forms. The study applies quantitative methods to a secondary dataset from a rural household survey, including entropy-weighted indicators, cluster analysis, and SEM models. The findings suggest that social capital has complex and significant impacts on household livelihood strategies. Bridging and linking social capital promotes non-agricultural and wage-based strategies, while bonding social capital drives transfer-based strategies. The study highlights the importance of social capital as a complementary resource to other livelihood capitals, such as financial, human, and physical capital. These results have important implications for policy implementation aimed at supporting rural households and their livelihoods, especially regarding social capital’s interaction with other livelihood capitals. By understanding the complex relationship between social capital, other livelihood capitals and livelihood strategies, policymakers can design more effective policies that harness the potential of social connections to support rural households.

## 1. Introduction

Bourdieu was one of the earliest scholars to discuss social capital in the modern academic way [1]. His approach was designed around meso-level and macro-level analysis units. Bourdieu argued that there was a diference in social capital between individuals belonging to various classes in eco-social development [2]. Social capital concepts were used to embed livelihood studies through the dimensions of links and networks. It allows individuals to access resources they may not possess on their own. Social capital is categorized as bonding, bridging, or linking. Bonding social capital refers to relationships among individuals who share common characteristics and belong to identifiable groups like kinship or community groups. These networks tend to be dense, making it easier for participants to monitor each other, resulting in network closure [3,4]. The social capital concept is viewed as an intangible capital of a household. According to DFID framework, there are five types of capital of household including: natural, physical, financial, human, and social capital [5]. Alternatively, Winters included valuable assets as a separate type of capital [6]. Social capital is always a crucial capital or a pillar source to improve households’ livelihoods. Additionally, social capital can be an alternative institutional basis for maintaining community trust, which can decrease transaction costs. Compared to other assets, social capital is not traditionally studied within the field of economics, but rather originates from sociology and spreads to other social science disciplines, becoming an interdisciplinary research topic. Social capital is increasingly being recognized as an important factor in promoting sustainable livelihoods for households. Furthermore, social capital replaces other institutions to ensure trust, community principles, and thus promotes social development, reducing transaction costs [1,7].

The sustainable livelihoods analysis framework DFID emphasizes that social capital is not only a livelihood asset that promotes sustainable livelihoods for households, but also an important factor that creates opportunities for livelihoods and promotes other livelihood assets of the household. Empirical evidence from different countries shows that households’ static and dynamic livelihood strategies are influenced by livelihood assets, of which social capital has become an important direct or indirect driving force in promoting livelihood strategy changes [8–12]. The “dynamic” nature of social capital, with its three forms (bonding, bridging, and linking) of social capital, has different characteristics [13–15]. Studies have found that bonding social capital not only supports to change livelihood strategies in the vunerable contexts, but also enhance specific changes in livelihood strategies such as migration, livelihood sensitivity [16], or in challenging livelihood contexts [17,18]. In a study by Morrison (1980), social capital with well-connected political resources can fill the gap in natural capital. This helps stabilize household livelihood strategies when social capital links are higher [19]. In previous studies using variables representing different forms of social capital, not only the stability of livelihoods but also the satisfaction of households increased [20,21].

In Vietnam, social capital often refers to the resources and relationships that people have through their social networks and community connections [22,23]. Previous studies showed that social capital can also provide emotional and social support during difficult times [24–27], or higher levels of linking social capital mean non-economic benefits for household livelihoods [28]. However, from of author’s point of view, few studies in Vietnam focused on the role of social capital and its interaction with other livelihood capitals on livelihood strategy. In a context of rapid change and many challenges for households, urbanization changes traditional family and village structures, international integration brings in new cultural values, and technology and the new living contexts [29–33]. Therefore, social resources and relationships may become important for improving household livelihoods, particularly in times of hardship or disaster. On the other hand, from theoretical perspective, through social capital, people are able to access support and resources (livelihood capitals) that they might not otherwise have access to.

To evaluate those effects, the regression appoaches are limited to estimate simultaneous both direct and indirect effects of factors. Meanwhile, the structural equation model (SEM) has many advantages to estimate and illustrate the complex relationships between variables, including both indirect and direct effect. That is suitable for evaluating the role of the dimentions of social capital on livelihood strategy. To our knowledge, few studies evaluate the dimentions of social capital on livelihood strategy of household, particular in the interaction effects between social capital and other livelihood capitals. So, this study aims to examine the role of social capital in different forms and its interactions with other livelihood capitals on household strategy. The study is divided into four parts: the topic and paper goals are describe at section introduction, the author reviews the theoretical foundations of social capital forms and its role in direct and indirect impacts on livelihood strategies at the theoretical foundation, the “Data and econometric approach” presents the data approach and quantitative methods, the “results and discussions” section presents the research results and discussion, and finally the author provides conclusions and policy implications.

## 2. Theoretical foundations and literature review

### 2.1 Bonding-bridging and linking social capital

According to Durlauf & Fafchamps’ paper, the social capital approach in economic studies has developed from Coleman, Lin to Putnam, Evans [3]. These scholars have focused on the role of social capital in shaping economic outcomes, such as household livelihoods. Meanwhile, Woolcock & Narayanhave proposed four perspectives to understand social capital: the public perspective, the institutional perspective, the network perspective, and the synthetic perspective [13].

A. Coleman argued that "Social capital is defined by its functions". Social capital is different from other types of capital such as physical capital or financial capital. Social capital is intangible and accumulated through the connections or relationships between individuals and organizations [34,35]. Coleman identified three forms of social capital: (1) The first form is based on the exchanges, expectations, and trust of social structures. Social capital can be built when individuals or groups engage in mutually beneficial exchanges or when they have shared expectations or trust in one another. (2) information channels—Individuals or groups with social capital are able to access information and knowledge through their social networks that they might not have access to otherwise. This can provide them with a competitive advantage in the marketplace, (3) norms and effective sanctions—Individuals or groups with social capital are able to access information and knowledge through their social networks that they might not have access to otherwise. This can provide them with a competitive advantage in the marketplace.

Portes figured out that social capital originates from two sources: the positive aspects of social capital, such as equal exchange and trust enforcement. These benefits can include social control through norms and laws, family support, and direct access to network connections. However, the negative consequences of social capital can include limited access to opportunities for those outside of networks, restrictions on individual freedoms [1].

Granovetter and Putnam focused on strong and weak ties in their studies, defining two forms of social capital: bridging social capital and bonding social capital [36]. Meanwhile, from the connections in social networks, especially weak ties, Lin argued that one of the important characteristics of social capital is to establish the flow of information in networks [2]. Burt, with his theory of structural holes in social capital, contributed significantly to a characteristic of social capital, which is the structural holes in the group forming social capital based on structural holes [37].

Grootaert & van Bastelaer divided social capital into three levels (micro, meso, macro) with two forms in structure perspective and cognitive perspective. The structural form of social capital is the foundation for sharing information and community activities as well as decision-making through roles and social networks, supplemented by laws, procedures, and precedents. Meanwhile, the cognitive form of social capital is expressed through norms, values, beliefs, attitudes, trust, and intangible social values [38].

According to Potapchuk, Crocker, & Schechtersocial capital is defined as links that brings members of community together [39]. From the theoretical perspectives to empirical studies, the World Bank research team emphasizes the "dynamic" nature of social capital with three forms (bonding, bridging, and linking), which also creates a consensus on the general forms of social capital [13–15,40]. Specifically, social capital has three levels based on close connections:

(i) The first level is bonding social capital, which includes direct relationships such as family, friends, and neighbors. This is a connection based on strong connections between individuals and direct interaction with each other.
(ii) The second level is bridging social capital, which includes more distant relationships such as colleagues, organizations, and similar links.
(iii) The third level is linking social capital, which are connections to public officers, especially political connections.

### 2.2 The role of social capital on household livelihood strategies

Narayan & Pritchett modelized the influence of social capital on household welfare in Tanzania. The authors found that the role of social capital is even more significant than the impact of education and tangible assets of the household. They emphasized the role of social capital at the village and community level, where households with higher social capital tend to be more innovative in their livelihood activities and agricultural production. Local organizations and associations play a critical role in 3 main pillars: information sharing, reducing opportunity costs, and a platform for consensus decision-making [41].

Ellis pointed that rural households/individuals are more concerned with livelihood strategies than net outcomes from income sources [42]. Furthermore, the author also argues that households’ living is achieved through utilizing income sources (money and similar kinds), social institutions, relationships, and property rights to support and maintain the common standard of living. So, social and family connections play an important role in households’ livelihood strategies.

Some studies have focused on measuring household livelihood sensitivity. The results indicate that the sensitivity of livelihood strategies is affected by household livelihood capital factors and the characteristics of the household’s geographic location. Among these factors, social capital and financial capital are catalysts for pursuits and changes in household strategies. Additionally, the impacts of these livelihood capital types are also dependent on the community or geographic location where the household lives [43,44].

Mogues studied the impacts of social capital forms on households’ living when households face shocks or risks in their livestock assets. The author emphasizes the role of households’ social capital in protecting livestock assets. However, social capital is not a factor that reduces the impacts of shocks or risks that households face [16]. In rural household livelihoods, Bebbington figured out the importance of social capital, although he emphasized that it is more difficult to understand than other types of capital due to its intangible nature. Furthermore, the author showed that social capital is the most critical capital for rural households to pursuit sustainable livelihoods [45]. Winters et al. also found that social connections have the significant impact on households’ participation in non-agricultural activities. This is one of pioneer studies on the diversification of livelihood activities in rural areas and farmers [46]. Livelihood strategies based on migration have also become an important topic. Studies have shown that migrant networks are established to share information and have a significant impact on potential migrants. Potential migrants can access to resources for helpful information, transaction costs, job oppoturnities from relatives, friends and their networks [47–49].

Recent studies have shown that social capital has stronger connections when households face different livelihood contexts and determine the ability to convert household livelihood strategies, According to Alemu in rural South Africa, households have high levels of social capital, which makes it easy for them to change their livelihood strategies and increases their chances of achieving higher incomes [50]. In addition, social capital and financial capital play an intermediary role in connecting and promoting non-agricultural livelihood activities as a solvent [51] or the role of network member can shift the chance of pursuiting the non-farm strategy[23,52].

In addition to the direct effects of social capital on livelihood strategies, DFID framework also emphasizes the indirect impacts of social capital on livelihood strategies through other forms of livelihood capital. Fafchamps and Gubert found that risk information is often shared within networks, which are mainly related to kinship, while economic relationships have no statistically significant impact on the sharing of such information [5,53]. Morrison identified political connections provide more benefits and conditions for promoting household livelihood activities. Additionally, households with more education or experience and better political resource connections can compensate for the shortage of land area in the village [19]. Recently, researchers analyzed an interaction framework between social capital and four other types of household capital to determine the channels of impact on household livelihood [22,23,54]. The authors emphasized that the revised analytical framework based on adjusting the livelihood analysis framework has the interaction of social capital with the other four types of capital is conveyed through three forms of social capital: trust, norms, and networks.

### 2.3 A proposed framework

**Fig 1:**
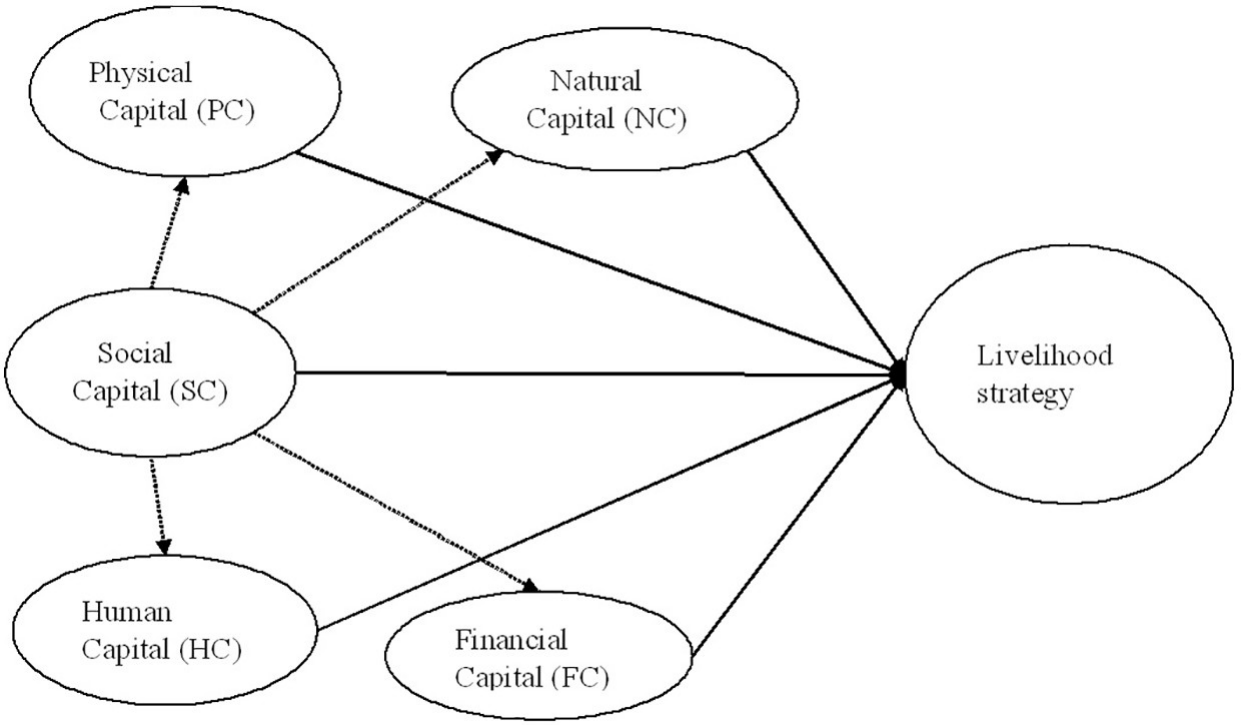
The proposed framework.

The proposed research analysis framework at Fig 1 still relies on the core principles of the DFID sustainable livelihoods analysis framework. Livelihoods are defined based on the sustainable livelihoods analysis framework, which includes three types of tangible assets: natural capital, physical capital, financial capital, and two types of intangible assets: human capital and social capital. Social capital not only has a direct impact but also an indirect impact on livelihood strategies through other forms of livelihood assets. Therefore, the research aims to test hypotheses on both the direct and indirect impacts of different forms of social capital on household livelihood strategies.

## 3. Data and econometric approach

### 3.1 Data and variables

This study uses the secondary dataset VARHS (Vietnam Access to Resources Household Survey). It is conducted to approximately 2600 households in 12 provinces. In this study, the author filtered and manipulated variables at 5 waves of survey with more than 10000 observations. Stata software is utilized for data manipulation and econometric model estimation.

Dependent variable: Livelihood Strategy. Livelihood strategies are the core point in household livelihood activities to achieve livelihood goals. The choice of livelihood strategies is determined by five types of livelihood capitals. Livelihood strategies in this study are measured based on an income approach using the Cluster analysis (K-means technique). Cluster analysis is widely used by researchers to construct composite indicators or to evaluate and classify groups based on household asset or income indicators to determine household livelihood strategies [17,55,56].

Independent variable:

**Social capital** (SC): The research proposed to examine three forms of social capital of households: Bonding, Bidging, Linking. Measuring through these three forms of social capital helps to accurately identify the dimentions of household social capital [13–15]. Bonding and bridging social capital are two forms based on network connections with a fundamental difference in the close connection characteristics between the group and related relationships [57]. To measure these two forms, the author proposes the evaluation of households’ perceptions of the cluster-bridge items. It is calculated by the weighted entropy methodology based on the level of importance of connections between households and organizations. Linking social capital is determined based on the level of connection of the household’s public organization network. The level of public/political connections is closely related to the number of connections and the level of position (with position in the public organization: 2, without position: 1).
**Human capital (HC), Physical capital (PC), Natural capital (NC), and Financial capital (FC)** are measured by many indicators. Authors aggregated each livelihood variable using entropy-weighted values based on these indicators. All indicators are listed in Table 1 and S1 Appendix.

**Table 1:** Livelihood capital and its indicators.

Livelihood Capital	Observed Variables	Indicator at database
Social capital Forms	Bonding-Bridging Social capital (SC-Bonding)	Number of members participating in organizations.
	Linking social capital (SC-Linking)	Number of organization connections
Human capital	Household size	Total household members
	Average Age of Household members	Calculate from member age
	Average Education of Household members	Calculate from member education years
Physical Capital	Asset index	Number of household assets
	Asset value index	Total value of household assets
	Housing area	Housing area
Natural Capital	Land area	Household’s land area
	Total value of land areas	Total value of Household’s land area
Financial Capital	Number of income sources	Number of income sources
	Saving value	Saving value
	Income per capita	Income per capita

### 3.2 Entropy methodology

The entropy method is used to integrate representative indicators for the types of livelihood capital of households that are derived from observed variables with weights calculated through this method to achieve higher reliability and accuracy[58,59]. The steps to compute weights are normalization of calculation value, determination of the ratio of household i of jth indicator, calculation of the Entropy operator of the jth indicator, and the calculation of the Entropy weight for each indicator.

1. Normalization of calculation value (*Fij* is original value of indicator j of household i)
$$Xij=\frac{Fij-min(Fij)}{Max\left(Fij\right)-min(Fij)}$$
2. The Probability of indicator j at household i—Pij:
$$Pij=\frac{Xij}{\sum_{1}^{n}Xij}$$
3. Entropy operator of indicator j:
$$Ej=(-1/lnn)\sum_{1}^{n}\left(Pij*lnPij\right)$$
4. Entropy weighted of indicator j:
$$wj=(1-Ej)/\sum_{1}^{m}\left(1-Ej\right)$$

Livelihood capitals are calculated using an entropy-weighted sum of their respective indicators, as explained in the appendix which outlines the measurement variables.

Consequently, the livelihood capital (F) is measured by: $F=\sum_{1}^{i}\left(Fi*wi\right)$, example: Human Capital index, $HC=HC={HC}_{1}*w_{HC1}+{HC}_{2}*w_{HC2}+{HC}_{3}*w_{HC3}$

### 3.3 Econometric methodology

#### 3.3.1 Cluster analysis

Cluster analysis is an approach that has been widely to assess and classify groups based on household asset or income indicators in order to determine the livelihood strategies of households [17,55,56]. The K-means clustering technique is defined by minimizing the variance or the sum of squared correlations within clusters (WCSS—within-cluster sum of squares). The K-mean approach uses Euclidean distance to measure the distance between data points. Given a sample of n observations that can be partitioned into n distinct clusters, K-means technique separates the n observations into k clusters f = (f1,f2,..fk) by minimizing the correlations within each cluster. Equation 1 describes the formula of K-mean approach:

$$arg_{f}min\sum_{i=1}^{k}{\sum_{x\in fi}\left|\left|x-\mu \right|\right|}^{2}=argfmin\sum_{i=1}^{k}\left|f_{i}\right|Varf_{i}$$ (1)

With: k the number of optimal clusters, i: represents each cluster, from 1 to k. ||*x* − *μ*||^2^: the squared Euclidean distance between a data point ’x’ and the centroid ’μ’. *Varf*_*i*_ show the variance of cluster i.

In this paper, K-mean approach is applied to determine the livelihood strategies of Vietnam rural households. The author uses 3 indicators to determine the number of household strategies: the proportional reduction of error (PRE) coefficient [60], lower bound technique (LBT) and Calinski-Harabasz pseudo-F index [61,62], which are presented at S2 Appendix.

#### 3.3.2 SEM model

In this paper, the author applied structural equation modelling (SEM) to estimate the interactions between social capital dimentions with other livelihood capitals. SEM is used to apply simultaneous equations to test the complex hypothesis in econometrics. The SEM model is often estimated the general regression with the mixed effects, including the limited denpendent variable such as logistic, poisson, multinomial logistic, and other models. For this paper’s model described at Fig 1, the SEM approach estimates simultaneous two models: structural model and measurement model. The structural model presented below illustrates the connections between the exogenous variables and the endogenous variables with accompanying model fit indices. In the measurement model section, the model fit, and error covariance are specified. Through the hypothetical examination, the author determines the direct and indirect impacts of social capital forms [63,64]. The goodness to fit of SEM model includes LogLikelihood, Akaike’s information criterion (AIC) and Bayesian information criterion (BIC). All models are estimated by robust estimation.

## 4. Results and discussions

### 4.1 Description of vietnam rural households

Table 2 showed that the social capital dimention is based on the bonding-bridging aspect is the clear and stable connection when the average reaches 0.11. The largest bonding-bridging social capital reached 0.44. The social capital dimention based on the linking aspect has continuously increased from 2008 to 2018, with an average level of 0.62 increasing to 1.04. However, the standard has also undergone significant changes, and the gap in social capital between households is increasingly widening.

**Table 2:** Indicator description.

Livelihood Capital	Indicators/ observed variable	min	mean	max	Entropy weighted value	Mean
Social capital	Bonding-Bridging (SC_Bonding)					.017
	Linking (SC_linking)					0.06
Human Capital	Household size	1	4.24	9	0.3	0.002
	Average age of household members	13.83	39.34	87	0.37	
	education	1.67	8.29	12	0.33	
Physical Capital	Housing area	4	77.92	230	0.37	-0.7
	the number of assets	0	5.49	12	0.07	
	Total asset value	0	16551.94	913700	0.56	
Natural Capital	Land area	15	6192.08	49440	0.49	-0.1
	Land value	0	111000	3325000	0.51	
Financial Capital	Income sources	1	3.43	6	0.03	-0.03
	Saving value	0	27724.98	350784	0.95	
	Income per capita	-60338.7	21748.2	141250	0.01	

On average, a household often have more than 4 members, and this has been stable throughout the survey years. The overall average age of the households was relatively stable at around 40–41 years old. Median of households had an average age of 37 years old or above. One of the important aspects of human capital is educational attainment. In terms of the general educational level of households, the average education index has slightly increased from 7.7 in 2008 to 8.26 in 2018. The educational attainment gap between households is relatively low. Natural capital is observed by three indicators: housing area, the number of assets, and the value of assets. The housing area of farm households has significantly increased from 2008 to 2018. Along with the increase in the housing area index, the number of assets also increased relatively from an average of 4.74 assets in 2008 to 5.78 assets in 2018. Furthermore, the total average value of assets of households in 2018 was 31.8 million VND, which is significantly higher than in 2008 (whether adjusted for inflation or not).

With agricultural activities, natural capital is a crucial factor in production activities. Statistics on the land area of households show that, on average, a household has over 6324 m2 (equivalent to about 0.63 hectares). The distribution is quite skewed as the median value is only 0.5 on the average value, and the standard deviation is much higher than the average value. This indicates a large disparity in natural capital among households. The financial capital of farm households is assessed based on three main indicators: the number of income sources, savings, income per capita. On average, households had about 3.5 income sources in 2018, implying that they usually have 3 to 4 income sources. This level has increased significantly compared to 2008, which is one of the indications that households have significantly diversified their income sources. The average statistics show that the average savings value of households is more than 27 million VND, with a significant difference between households. The 2018 statistics show that on average, households had a per capita income of around 30 million VND, of which many households had negative per capita income due to financial losses in the year. The average income of households has increased significantly compared to 2008 (even after adjusting for inflation), while the income inequality between households remains relatively high.

### 4.2 Vietnam rural household livelihood strategies

Using the Kmeans clustering approach with eight identified income sources, the results at Table 3 show that four clusters are the optimal number based on various indicators, with up to six different indicators suggesting that four clusters are the most optimal allocation. Based on the cluster allocation, statistics on the average contribution of each income source to each cluster show significant differences in the characteristics of income sources for each cluster.

**Table 3:** Kmeans results and livelihood strategy.

Livelihood Strategies	Wage	Agri-cultural	Common resource	Non-agricultural	Rent	Sell property	Transfer from private	Transfer from public	others
Non-agricultural-based	9.11%	8.54%	0.68%	**71.14%**	0.58%	0.93%	3.80%	2.65%	1.29%
Agricultural-based	9.78%	**68.47%**	3.36%	2.26%	0.23%	0.57%	4.02%	3.27%	0.69%
Wage -based	**73.37%**	12.12%	1.27%	3.14%	0.43%	0.49%	4.04%	3.32%	0.71%
Transfer-based	7.82%	11.35%	3.58%	2.62%	1.77%	4.38%	**32.16%**	**31.53%**	1.98%

In the initial livelihood cluster, the predominant sources of income are derived from non-agricultural activities, contributing an average of approximately 72% to the total income. Meanwhile, alternative sources of income remain relatively limited. Consequently, this livelihood strategy is named as the **non-agricultural-based strategy (LS1)**. The second livelihood cluster has the highest average contribution from agricultural activities, with over 68% of income sources from agriculture, while the contribution from other sources of income is so small. Thus, the livelihood strategy associated with cluster three is named the **agricultural-based strategy (LS2)**.

In cluster three, households have income sources from various sources but mainly receive from wages, with an average contribution of over 73%. Therefore, the livelihood strategy associated with the third cluster is named the **wage-based strategy (LS3)**. Finally, in the fourth cluster, two income sources from transfer sources contribute an average of over 32%, indicating that the main income sources for households receiving transfers include personal transfers and public supports/transfer. Therefore, the livelihood strategy associated with this cluster is named the **transfer-based strategy (LS4)**.

### 4.3 Econometric results

To assess the impact of social capital on household livelihood strategies based on 5 types of livelihood capital, the author applied the Generalized Structural Equation Model (GSEM) under robust estimations. The results at Table 4 showed that the estimated models were appropriate and statistically significant in the analysis.

**Table 4:** GSEM results.

Variable	Livelihood strategy choice (vs based strategy: LS2 –agricultural -based strategy)			Social capital effects on other capitals	
	LS1	LS3	LS4	SC_Bonding	SC_Linking
SC_Bonding	-0.124*** (0.004)	-0.064** (0.039)	0.106*** (0.002)		
SC_Linking	-0.078** (0.047)	0.031 (0.249)	0.033 (0.317)		
HC	0.622*** (0.00)	1.015*** (0.00)	1.273*** (0.00)	0.127*** (0.00)	0.051*** (0.00)
PC	1.116*** (0.00)	0.526*** (0.00)	-1.216*** (0.00)	0.049*** (0.00)	0.043*** (0.00)
NC	-1.255*** (0.00)	-1.31*** (0.00)	-1.319*** (0.00)	0.018*** (0.00)	0.024*** (0.00)
FC	0.293*** (0.00)	0.047 (0.446)	0.336*** (0.00)	0.063*** (0.00)	0.054*** (0.00)
_cons	-0.89*** (0.00)	0.295*** (0.00)	-0.715*** (0.00)		
var(e.HC)	0.182	Model Summary: Log pseudolikelihood = -34884.917 df: 37 AIC = 69843.83 BIC = 70104.83			
var(e.PC)	0.143				
var(e.NC)	0.182				
var(e.FC)	0.784				

Note: The t-statistic is in parentheses;

*, **, *** indicate significance at the 10%, 5%, and 1% levels, respectively.

Social capital in the form of bridging–bonding is a driving factor for households to move beyond agricultural livelihood strategies, particularly toward non-agricultural livelihood strategies. The higher the level of social capital in the bridging dimention, the more it encourages households to choose or transition to non-agricultural livelihood strategies. This result confirms the positive influence of the bridging social capital form on the selection of non-agricultural livelihood strategies by households in a positive trend through social connectivity relationships.

In addition to non-agricultural livelihood strategies, wage-based strategies are also livelihood strategies influenced and promoted by bridging social capital. In other words, as a household’s bridging dimention significantly increases, the household tends to choose wage-based strategies not only for themselves but also in comparison to other strategies. Results from the quantitative models show that bridging social capital promotes wage-based strategy even in comparison with other livelihood strategies (except non-agricultural livelihood strategies).

While traditional agricultural or transition livelihood strategies are pursued by households with social capital in the bonding dimention, in comparison to other livelihood strategies, the bridging social capital form and linking social capital promote wage-based strategy and non-agricultural-based strategy. A particular feature in the linking social capital form is the move away from non-agricultural and dominant agricultural livelihood strategies.

### 4.4 Discussion

#### 4.4.1 Direct effects

By synthesizing the impact of livelihood capitals on the livelihood strategy model, the direct role of two social capital variables on the livelihood strategies in Vietnam are revealed. The results at Table 3 show the direct influence of social capital, with three forms of social capital through two variables measuring social capital, having significant differences and absolute values in the impact of social capital relatively. In this aspect, the Sustainable Livelihoods Framework [5] emphasized the complex impact of social capital, which may have both positive and negative effects through networks or organizations. This is also one of the advantages of analyzing social capital based on the characteristics of its forms, thus more clearly distinguishing the impact mechanisms of social capital.

Table 5 shows that the higher the bridging social capital of a household, the more dominant livelihood strategies are selected, as they are non-agricultural-based strategy and wage-based strategy. Moreover, the more connected the social capital of a household, the more sustainable its livelihood strategy. This implies stability in household livelihoods through the selection of reasonable strategies and achieving their livelihood goals. This reflects the weak relationships in the connections based on networks that the weak tie theory and the structural hole theory refer to. Relying on the aggregated network structure of the household, where weak relationships become more significant, the higher the bridging social capital, the interaction and information exchange process provides individuals with new/different information sources, enabling households to access more new/different information, promoting advantages or seizing opportunities in selecting appropriate non-agricultural-based or wage -based strategies. In another aspect, choosing non-agricultural-based strategies differs significantly from traditional agricultural livelihoods; thus, only households with different information due to bridging relationships in social capital can effectively pursue these strategies. The practical pursuit of non-agricultural-base strategy through the benefits of network connections has also been evidenced in many studies worldwide, Winters confirmed the significant impact of social relationships on the ability to participate in non-agricultural activities in Mexico [6], and Fang et al. confirmed that social capital plays a role in connecting and promoting non-agricultural livelihood activities [44]. The sustainability of livelihoods when facing the vulnerable context is the cooperation and connection activities [65–67]. In the meantime, weak tie-based relationships increase opportunities for farm households to participate in labor markets to pursue livelihood strategies based on weak links between individuals from different organizations/groups, overcoming the structural holes of social capital [36].

**Table 5:** Direct effects of social capital.

Variable	Livelihood Strategy Selection					
	LS1 vs LS2	LS3 vs LS2	LS4 vs LS2	LS1 vs LS3	LS4 vs LS3	LS4 vs LS1
SC_Bonding	-	-	+		+	+
SC_Linking	-			-		+

Furthermore, bonding form is a factor that encourages households to pursue remittance-based livelihood or transfer-based strategy. The high level of bonding social capital demonstrates a strong connection between farm households and strong ties, such as relatives, family members, rather than different relationships. One advantage of bonding social capital is the reduction of risks and better support from strong relationships in the face of livelihood fluctuations [20,23,68]. At the same time, the transfer-based strategy depends heavily on remittance income from individuals and communities; in other words, part of the income is received from migrated household members. Through the support of high bonding social capital, the potential to reduce risks and costs when implementing livelihood transition strategies and pursuing decisions such as migration is increased [69].

In comparison with bonding and bridging social capital, linking social capital has a significant difference. Linking capital inherently has a hierarchical network in society, especially in traditional agricultural societies influenced by Eastern culture and Confucianism, such as Vietnam, China, South Korea, or Japan, where the degree of hierarchy in social values has a significant impact. From Table 4, the direct impact of linking social capital shows a trend away from non-agricultural-based strategy. Consequently, when choosing a livelihood strategy and comparing it with other livelihood strategies, wage-based strategy is the preferred choice, and non-agricultural-based strategy is often overlooked when households have high linked social capital. The connections within high linking dimention help rural households gain advantages in social positions, which allows them to pursue wage -based strategy more easily [28,54].

#### 4.4.2 Indirect effects

In addition to the direct impact on the pursuit of household livelihood strategies, social capital interacts and influences other types of livelihood capital, affecting strategies and shifts in household livelihoods. Quantitative model results at Table 4 confirmed the impact of social capital on other livelihood capitals of farm households. Both Bonding-Bridging social capital and linking social capital have positive impacts on other livelihood capitals, which implies that bridging form has a negative impact on other livelihood capitals (and bridging form has a positive impact). Thus, at Table 6, in addition to the direct impact of social capital on farm households’ livelihood strategies, there is an indirect impact through its influence on other sources of livelihood capital.

**Table 6:** Direct-Indirect-Total effects of social capital.

Biến	Effects	LS1 vs LS2	LS3 vs LS2	LS4 vs LS2	LS1 vs LS3	LS4 vs LS3	LS4 vs LS1
**SC_Bonding**	Direct	-	-	+		+	+
	Indirect	+	+	+	-	-	-
	**Total**	**+**	**+**	**+**	**-**	**+**	**+**
**SC_Linking**	Direct	-			-		+
	Indirect	+	+	-	+	-	-
	**Total**	**-**	**+**	**-**	**-**	**-**	**+**

The results showed that human capital plays an extremely important role in pursuing household livelihood strategies, where the advantage of livelihood strategies, through the impact of human capital, tends to not select agricultural strategies. Through human capital, social capital promotes non-agricultural-based strategies, including wage-based and transfer-based strategies, which are strongly encouraged when jobs in these sectors have higher technical requirements, relatively narrow fields, and relatively high incomes [59]. These results confirm the hypothesis of the indirect impact of social capital on human capital and its influence on the choice and comparison of household livelihood strategies. This also reaffirms the findings of previous studies on the stimulating influence of social capital on human capital, and its impact on the pursuit of household livelihood strategies [53,54,70].

The indirect impact of social capital on physical capital and its influence on the choice and comparison of household livelihood strategies is also significant. The more physical capital a household has, the less likely it is to change its livelihood strategy, whereas a shortage in physical capital encourages households to change their livelihood strategies. One of the main reasons for this is that rural households with high physical capital tend to have large valuable assets, which generally meet their livelihood needs, so they choose not to change their livelihood strategies to minimize risk [71]. Moreover, this also confirms that when there is a shortage of physical capital and high social capital, implying that through physical capital, farm households rely on high social capital to change their livelihood strategies towards more sustainable livelihood strategies.

One of the key characteristics of farm household livelihood capital, particularly in Vietnam, is natural capital. Agriculture, farmers, and rural areas are closely linked to natural capital indicators, most importantly land, which is closely related to agricultural production activities. This study confirms the indirect impact of social capital to pursue agricultural livelihood strategy. This is consistent with the results of many other studies that confirm the influence of natural capital, such as land, and social status, in promoting household livelihood diversification [72–74]. Morrison has shown that social capital with better political connections can fill the gaps in natural capital, helping to stabilize household livelihood strategies when linking social capital is higher. In previous research using variables representing various forms of linking social capital, not only livelihood stability but also farm household satisfaction was increased [20,21]. In Vietnam, higher linking social capital implies tighter political connections, which are often tied to public servants or political network.

The indirect impact of social capital through the financial capital channel is significant when households have both high financial capital and social capital (through both bonding form and linking form), promoting the pursuit of non-agricultural-based and transfer-based livelihood strategies. This is explained by many studies through increasing income by increasing financial assets or promoting non-agricultural activities requiring relatively high financial investments compared to other strategies [26,75,76].

## 5. Conclusion

In summary, the direct effects of social capital, in its various forms, have significant implications for the livelihood strategies and sustainability of rural households in Vietnam. Bridging social capital promotes non-agricultural-based and wage-based strategies, bonding social capital encourages transfer-based strategies by reducing risks and providing better support, and linking social capital drives households towards wage-based strategy. In addition, this research highlights the significant and complex role of social capital in shaping household livelihood strategies. Social capital has both direct and indirect impacts on the pursuit of various strategies, influencing other types of livelihood capital such as human, physical, natural, and financial capital.

In Vietnam, most households live in rural areas, their livelihood and their well-being face to many challenges in livelihood contexts. Vietnam is one of the most vulnerable areas from climate change, rising sea levels. That affects directly rural households’ livelihoods. From households’ perpective, they have lack of livelihood capitals or livelihood sources to pursue of sustainable livelihood strategy. However, the difficulty of livelihood policies also enhance the capacity of household in livelihood capitals. The policy designation faces the difficulty to support households accessing financial resources or natural resources. Meanwhile, human capital often depends on the self-development of household. Consequently, by recognizing the interconnections between social capital and other livelihood capitals, policymakers and development practitioners can design interventions and support systems that enhance the adaptive capacity and resilience of farm households in the face of economic, social, and environmental changes. Information channels can access and promote effective livelihood strategies for farm households and leverage various forms of social capital to enhance the effectiveness of other sources of livelihood capital, particularly the roles of bridging and linking social capital. This study can delve into the indicators of livelihood capitals for exploring the direct impact of the dimensions of livelihood capitals on households’ livelihood. In addition, future studies should focus on the livelihood outcome or household resillience.

## Supporting information

S1 Appendix Measurement of livelihood capitals. (DOCX)

S2 Appendix Kmeans Estimation indicators. (TIF)
