# Correction: The role of social capital in shaping livelihood for rural Vietnamese households

**DOI:** 10.1371/journal.pone.0299867

**Published:** 2024-02-27

**Authors:** Huynh Ngoc Chuong

There are errors in the Funding statement. The correct Funding statement is as follows: The funding for this research was provided by the University of Economics and Law (UEL), Vietnam National University Ho Chi Minh City (VNU-HCM). The funder had no role in study design, data collection and analysis, decision to publish, or preparation of the manuscript.
