# Supplementary material for: The role of social capital in shaping livelihood for rural Vietnamese households
Source: PLoS One. 2023 Dec 14;18(12):e0295292. doi: 10.1371/journal.pone.0295292 (PMC10721011; doi:10.1371/journal.pone.0295292)
Supplement: S1 Appendix — (DOCX) [file pone.0295292.s001.docx]

**Appendix 1: Measurement of livelihood capitals**

| Livelihood Capital | Observed Variables | Indicator at database | Description of livelihood capital measurement |
| --- | --- | --- | --- |
| Social capital Forms | Bonding-Bridging Social capital (SC-Bonding) | Number of members participating in organizations. | Calculate entropy weights based on the characteristics of participating organizations. |
|  | Linking social capital (SC-Linking) | Number of organization connections | Calculate entropy weights based on the characteristics of position in organizations |
| Human capital | Household size | Total household members | Entropy-weighted value is calculated from standardized variables |
|  | Average Age of Household members | Calculate from member age |  |
|  | Average Education of Household members | Calculate from member education years |  |
| Physical Capital | Asset index | Number of household assets | Entropy-weighted value is calculated from standardized variables |
|  | Asset value index | Total value of household assets |  |
|  | Housing area | Housing area |  |
| Natural Capital | Land area | Household’s land area | Entropy-weighted value is calculated from standardized variables |
|  | Total value of land areas | Total value of Household’s land area |  |
| Financial Capital | Number of income sources | Number of income sources | Entropy-weighted value is calculated from standardized variables |
|  | Saving value | Saving value |  |
|  | Income per capita | Income per capita |  |
