# Supplementary figures and images for: The role of social capital in shaping livelihood for rural Vietnamese households
Source: PLoS One. 2023 Dec 14;18(12):e0295292. doi: 10.1371/journal.pone.0295292 (PMC10721011; doi:10.1371/journal.pone.0295292)

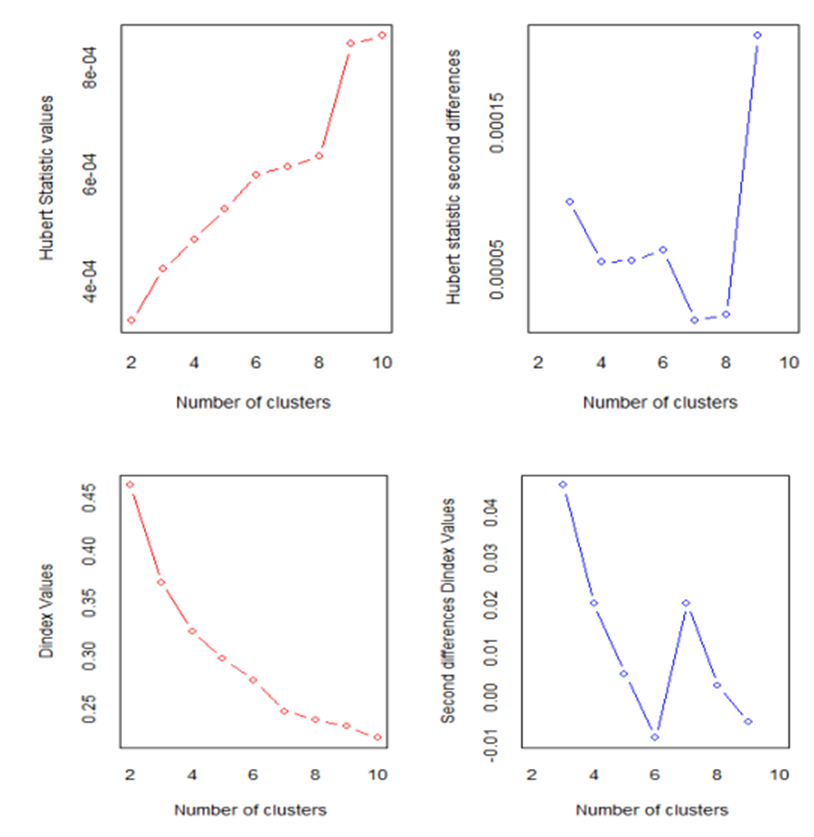

Supplement: S2 Appendix — (TIF) [file pone.0295292.s002.tif]
